# Frozen, Cold, or Cool? Chemical Assessment of the Effectiveness of Storage Conditions for Celluloid 3D Objects

**DOI:** 10.3390/polym15204056

**Published:** 2023-10-11

**Authors:** Christina Elsässer, Eva Mariasole Angelin, Peter Montag, Harald Hilbig, Christian U. Grosse, Marisa Pamplona

**Affiliations:** 1 Conservation Science Department, Deutsches Museum, Museumsinsel 1, 80538 Munich, Germany; c.elsaesser@deutsches-museum.de (C.E.); e.angelin@deutsches-museum.de (E.M.A.); 2 Chair of Non-Destructive Testing, Technical University of Munich, Franz-Langinger-Straße 10, 81245 Munich, Germany; grosse@tum.de; 3 PSS a Part of Agilent, Polymer Standards Service GmbH, In der Dalheimer Wiese 5, 55120 Mainz, Germany; pmddf@web.de; 4 Professorship of Mineral Construction Materials, Technical University of Munich, Franz-Langinger-Straße 10, 81245 Munich, Germany; harald.hilbig@tum.de

**Keywords:** three-dimensional celluloid, camphor, low-temperature storage, museums, ion chromatography, gel permeation chromatography, mock-ups, degradation, depth profiling

## Abstract

Preserving celluloid artifacts is challenging for museums, as this plastic is highly prone to degradation. Frozen, cold, and cool storage solutions are typically recommended for inhibiting the chemical degradation of celluloid. However, they are rarely implemented for three-dimensional celluloid (3D-CN) objects because low temperatures might cause irreversible effects (e.g., microcracking). This work presents the effects of four different storage temperatures (+23 °C, +13 °C, +9 °C, −15 °C) on the preservation of artificially aged 3D-CN mock-ups, aiming at understanding their effectiveness by measuring molecular weight distribution, camphor, and nitrogen contents after storage. Gel permeation chromatography (GPC) results showed that the least loss of camphor content and fewer polymer chain scissions happened at −15 °C, hinting that this temperature was the best for preservation. However, the heterogeneous nature of celluloid alteration, i.e., the development of degradation gradients in thicker 3D-CN objects (>0.5 mm), made it necessary to apply a novel sampling technique, which selectively considers several depths for analyses from the surface to the core (depth profiling). This depth profiling made monitoring the degradation evolution dependent on the storage conditions in the thicker mock-ups possible. This approach was also used for the first time to quantify the polymer chain scission, camphor loss, and denitration of historical artifacts, indicating a dramatic difference in the degradation stage between surface and core. The effectiveness of frozen storage on the chemical stability of 3D-CN after seven months could support museums to consider reducing the storage temperatures to preserve precious artifacts.

## 1. Introduction

Celluloid, a mixture of cellulose nitrate (CN) with up to ~33% weight of camphor as a primary plasticizer [1,2,3,4,5,6], is considered one of the least stable plastics in museum collections [3,7]. Many artifacts made of celluloid show dramatic signs of degradation due to the inherent material instability, which can lead to material collapse through main polymer chain scissions, NO_x_ emissions (denitration), and loss of camphor [3,5,8,9,10,11,12,13,14].

Generally, one can divide plastic degradation into two phases [3]. The first degradation may occur during plastic manufacturing, such as molding and extrusion. Indeed, the plastics can be subjected to high temperatures during processing, providing opportunities for thermal and oxidative degradation. The second starts right after production when the plastic is continually exposed to air, moisture, light, and heat, also known as environmental weathering. Even though the manufacturing process might cause the first alteration, environmental factors typically induce the most significant changes in the degradation state of objects after entering the museum. The condition state of three-dimensional celluloid (3D-CN) objects is usually divided into three states by conservators [15,16] through visual inspections and basic assessment of acidic emissions with suitable indicator papers [15,17]. State I is classified as visibly in good condition without acid detection. State II is defined as a moderate condition, meaning that 3D-CN objects can visually show only slight changes, such as loss of transparency, yellowing, and minor deformations, but emit nitrogen oxides, which can react with moisture, forming the pair nitrous acid/nitric acid (HNO_2_/HNO_3_) [18]. State III is described as a severe condition referring to objects visibly exhibiting heavy signs of degradation, such as cracks and crazing, yellowing, deformations, and acid detection. Even though this classification gives a first impression of the overall condition of 3D-CN objects, it has some limitations, as the degradation extent in severe conditions is visibly non-uniform [3,5,6,10,13,17,19,20]. Several studies [5,6,13] showed that the condition state of severe 3D-CN objects could greatly vary, and degradation gradients in the width and depth typically develop, highlighting that severely aged objects will not present just one condition but rather a range of condition states.

Cool, cold, and frozen storage conditions are widely recommended for preserving CN cinematographic films and photographic materials (2D-CN) [9,21,22,23,24,25,26,27,28,29,30,31] because by lowering the surrounding temperatures, the rate of chemical degradation can slow down, prolonging their museum lifetimes. Below room temperature solutions also enhance the safety of 2D-CN collections. Indeed, 2D-CN are generally higher nitrated (nitrogen content: explosives >12–13%, films ca 12%; molded plastics 10.5–11.2%) [2] than 3D-CN and therefore are considered highly flammable materials [32]. Compared to 2D-CN, studies are seldom assessing the effectiveness and harmfulness of cool, cold, and frozen storage on 3D-CN objects [33,34,35]. Even though low temperatures slow down the chemical degradation of plastics, they can also lead to permanent dimensional changes and physical failure due to insufficient ductility [36,37,38]. In particular, 3D-CN objects are endangered from suffering such physical failure due to their thickness, shape, and various material combinations. Furthermore, the conservation community debates the substantial financial means required by these preservation strategies [31,39,40] and raises environmental concerns about energy consumption and CO_2_ emissions.

To the authors’ knowledge, only a few institutions have started investigations on the low-temperature storage of 3D-CN. The British Museum conducted pilot studies to examine the effects of freeze–thaw cycles, such as cracking and water condensation, on 3D-CN samples representing badges [41] and shadow puppets (project number: PR07406) [42] before transferring their collection to freezer storage. The Getty Conservation Institute (GCI) leads a project [15] on storage solutions for naturally aged cellulose ester objects, considering ambient, cool, cold, and frozen storage conditions. Preliminary results assessing the molecular weight (M_w_) distribution and nitrogen content showed no changes after six months, and further analytical results from longer storage time are upcoming [15]. Since the 1990s, the Canadian Museum of History (CMH) froze just some selected plastic objects and, from 2005 onwards, stored most of its 3D-CN collection in chest freezers [43]. The conservation scientists at the CMH plan to investigate the conditions of the frozen 3D-CN objects after more than 18 years of storage and to evaluate the effects of freezing accordingly [43]. In this context, a research project [44,45] was conducted at the Deutsches Museum (DM) that involved: (i) the production of 3D-CN mock-ups [16]; (ii) the selection of chemical characterization methods [5,14]; (iii) the optimization of artificial aging protocols for mock-ups to simulate degraded museum objects [16]; and (iv) the assessment of the effectiveness of four storage temperatures on the preservation of 3D-CN mocks. This paper is dedicated to the last step, the chemical investigation of moderately aged mock-ups shaped in three geometries (sheets, tines, and cylinders) after seven months of storage at +23 °C, +13 °C, +5 °C, and −15 °C.

In steps from (i) to (iii), the chemical characterization involved bulk analyses, which consider the whole depth of the mock-ups or naturally aged objects [5,14,16]. According to Mazurek et al. 2019 [13], those bulk analyses successfully characterized different conditions in areas of the same severely aged objects visually contrasting (degraded and cracked against intact) and verified the development of alteration gradients in width. Meanwhile, the authors [6] investigated the heterogeneous nature of celluloid, highlighting that not only is the sampling location of great importance due to the heterogeneous nature of the alteration but also degradation gradients can occur along the depths (surface to the core) of thick-walled objects. Bulk analyses are unsuitable for recognizing degradation gradients in depths because the results are averages of the materials resulting from the surface and core.

This work uses, for the first time, a multi-analytical approach that applies two sampling techniques to assess the effectiveness of cool, cold, and frozen storage conditions after seven months compared to room temperature (RT). The first sampling technique (1) utilizes the complete depths of a mock-up for the analyses (bulk), while the second (2) selectively considers several depths for analyses from the surface to the core (depth profile) in order to assess the temperature’s effect on preserving thick-walled mock-ups. In bulk (1), M_w_ and camphor content were assessed on all geometries (sheets, tines, and cylinders). In a depth profile (2), the M_w_, camphor, and nitrogen contents were measured for the cylinders and historical objects to characterize their spatial distribution and suggest a possible evolution from state II to state III, paving the way for future systematic research into the evolution of degradation gradients in the profile of 3D-CN objects.

## 2. Materials and Methods

### 2.1. Mock-Ups: Processing and Artificial Aging

Three mock-up geometries were selected for this storage investigation. The mock-up shapes were based on the celluloid artifacts surveyed at the DM: sheets (20 mm (L) × 20 mm (W) × 0.5 mm (D)), representing industrial plastic swatches, tines (50 mm (L) × 16 mm (W) × 2 mm (D)) recreating the geometry found in combs, and cylinders (30 mm (L); Ø = 10 mm), with a metal core (nickel silver rods, 2 mm in diameter (Gemmel Metalle)), referring to the geometry found in eyeglasses [38]. The authors describe the details of the mock-up processing and aging elsewhere [16]. In summary, the mock-up geometries were gained by cutting and processing prefabricated transparent colorless celluloid sheets (Incudo Clear Transparent Celluloid Sheet RF0425, 430 mm × 290 mm × 0.5 mm) purchased from Rothko and Frost™. The following processing method was applied to gain suitable thicknesses required for the tines and cylinders. The prefabricated sheets were cut in geometries following the contour of tines or cylinders and then layered to produce thick 3D-CN shapes. The precut geometries were softened in acetone (≥99.5% for synthesis, Carl Roth) for one day to ensure better cohesion between layers. The softened 3D-CN shapes were then transferred to a metal mold and altogether heated in the slap press (PCS II, COLLIN) for 5 (tines) or 15 (cylinders) minutes before molding at 25 bar for 8 min at 90 °C. The mock-ups were cooled in a water bath before removing the molds. The final surface treatments involved removing excess material around the mock-ups (flash) and polishing to complete the processing.

Thermohygrometric artificial aging in a fan-assisted dynamic climate chamber (MKF 115, Binder, Tuttlingen, Germany) was applied to accelerate deterioration processes in the newly prepared mock-ups and make them more comparable to naturally aged museum objects. The conditions of aging were as follows:

- Sheets: 70 °C and 75% relative humidity (RH) for 32 days;
- Tines: 60 °C and 75% RH for 17 days;
- Cylinders: 60 °C and 75% RH for 27 days.

A comparison with historical objects highlighted that those mock-ups represent moderately aged artifacts [16] depicting the characteristic degradation phenomena of celluloid in moderate conditions (state II), such as loss of transparency, yellowing, slight deformations, and off-gassing (acids detection).

### 2.2. Enclusures for Storage of Mock-Ups

**Figure 1:**
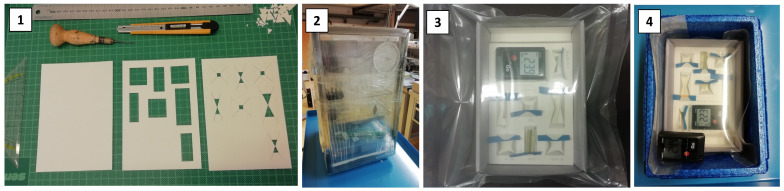
Sequence of steps before the storage of mock-ups: Selection and preparation of packaging materials (1); preconditioning of mock-ups and packaging materials (2); enclosing of mock-ups (3); and transferring enclosures in Thermo boxes to the tested storage conditions (4).

The selection of materials and preparation steps before storage was based on the experience gathered for freezing archival materials [46,47,48] and the latest recommendations and approaches in the freezer storage of 3D objects [15,49]. Figure 1 reports the different steps needed to prepare the mock-ups for storage.

Step 1: Selection and preparation of packaging materials. The materials required for the packing can be divided into three main categories according to their purpose. First, materials that have a clear protective function during storage (e.g., padding, stability, humidity buffer, water penetration); second, inserted equipment for monitoring purposes (e.g., data-logger, indicator papers); third, a sealable barrier film needed to create a uniform environment (i.e., closed environments with similar volumes) and therefore a comparable condition for each package at the beginning of the experiment. Mock-ups for all envisaged storage temperatures were packed and sealed. A detailed list of the materials and their specific function is given in Table 1. Each package hosted four mock-ups of each geometry. Due to limitations in the storage space, the mock-ups were arranged on two levels (Figures S1 and S2).

Step 2: The mock-ups and the packaging materials were preconditioned at 36% RH and 22 °C for 12 days in a desiccator. A graph for the temperature and RH in the desiccator can be found in the Supplementary Information (Figure S3). 

Step 3: Then, the mock-ups were removed from the desiccator and, as quickly as possible, mounted in the archival boxes (Figure S2) and sealed by barrier film. 

Step 4: The next day (around 12 h later), the sealed packages were enclosed in Thermo boxes (Kängabox^®^Epert Mini, FEURER Febra GmbH, Brackenheim, Germany) made of 1.5 cm thick expanded polypropylene and transferred to their specific storage condition.

### 2.3. Storage

Based on the authors’ previous work [38], which reflects on storage recommendations for 3D-CN and related comments given by conservation experts in an international poll, suitable temperatures for the cool, cold, and frozen storage tests were defined. At the same time, those temperatures were accessible in the DM and, therefore, realistic for other museum collections. Cool temperatures of +13 °C were achieved by an air conditioning system installed in a room for archival materials preservation at the DM, cold temperatures of +9 °C by a fridge, and freeze temperatures of −15 °C by a freezer. The sealed packages were also located in a lab environment with a yearly average of +23 °C, serving as a reference. The RH in the enclosures reached a maximum average of 42% (RT) and a minimum average of 33% (freezer). Detailed thermohygrometric diagrams during the storage are given in the Supplementary Information (Figure S4). The mock-ups were exposed to these four storage conditions for seven months, from July 2021 to February 2022. After this period, the Thermo boxes holding the sealed packages were removed from storage and left for acclimatization for 24 h at RT, before opening them. The mock-ups and indicator papers (see Section 2.4.) were photographically documented and then, until and between analysis (see Section 2.7, Section 2.8 and Section 2.9), kept in the dark at RT inside a safety storage cabinet (Q90.195.120, asecos^®^ GmbH, Gründau, Germany) with permanent filtered (active charcoal) air ventilation.

### 2.4. Off-Gassing Monitoring with A-D Strips

To monitor if the mock-ups were emitting acid vapors during storage, A-D strips (Tabel 1), acid–base indicators made of paper strips dye-coated with Bromocresol green indicator developed by the Image Permanence Institute (IPI), were integrated into the packaging system of the mock-ups. In the presence of acids, the indicator will change from blue, via green, to finally yellow, depending on the pH. Even though such A-D strips were designed for acetate films [24], they can also be applied to detect celluloid acidic emissions [15,17]. Because of their ease of application, these indicators are often used in the conservation community. After the storage time of seven months, the color change of those A-D strips was visually investigated to estimate their responses and connect the intensity of acid detection at different temperatures to the chemical characterization of corresponding mock-ups.

### 2.5. Ion Chromatography (IC)

Following the sample preparation proposed by Mazurek et al. [13], 5 mg of celluloid sample was dissolved in 1 mL acetone (acetone 99.5% for synthesis, Carl Roth GmbH + Co. KG, Karlsruhe, Germany) at 50 °C for one hour. The naturally aged samples in severe conditions did not dissolve in pure acetone; therefore, these samples were dissolved in 1 mL 7/3 (acetone:water) mixture at ca. 50 °C for two hours. The process of alkaline hydrolyses was conducted by adding 2 mL 1 N NaOH (Merck, Burlington, MA, USA) to the previously dissolved samples and heated for 2 h at 60 °C. Finally, 17 mL water was added for a final volume of 20 mL. Before analysis, the sample solution was 100-fold diluted (0.5 mL sample solution added to 50 mL water) and then measured by a Metrohm 940 Professional IC Vario Ion Chromatograph (Metrohm AG, Herisau, Switzerland) equipped with a set of Metrosep columns (pre-column Metrosep A Supp 5 Guard/4.0 and main column Metrosep A Supp 7-250/4.0). The columns were maintained at 45 °C, and the eluent was 3.6 mM sodium carbonate (Merck, Burlington, MA, USA) at a flow rate of 0.7 mL/min. Nitrite standard solution (Centipur^®^, Merck, Burlington, MA, USA) and the Anion multielement standard I and II (Centipur^®^, Merck, Burlington, MA, USA) were used as calibration standards. The theory/calculation summarized by Mazurek et al. [13] was used to calculate the nitrogen content. In contrast to [13], no previous plasticizer extraction was made in this research. Therefore, for each 5 mg celluloid sample, the respective amount of camphor measured by GPC was subtracted.

### 2.6. Gel Permeation Chromatography (GPC) for Determining the Molecular Weight

Based on the method by Kavda et al. [14], 5 mg of the sample was dissolved in 1 mL of N,N-dimethylacetamide (DMAc 99.5% HPLC grade, Alfa Aesar) with 0.5% *w*/*v* lithium chloride (LiCl ACS 99%, Alfa Aesar) and left to dissolve for 25–28 h, frequently manually shaken. Before injection, samples were filtered through a poly (tetrafluoroethylene) filter (PTFE, pore size 0.2 μm, diameter 25 mm) to remove insoluble material and impurities. Then, 30 μL of sample solution was injected by an autosampler into the GPC system (SECcurity2, PSS, Mainz, Germany). This system was equipped with a set of PSS GRAM columns (pre-column 10 μm 8 × 50 mm, 1 column 10 μm 30 Å 8 × 300 mm, and 2 columns 10 μm 1000 Å 8 × 300 mm) all sustained at 60 °C. The eluent DMAc/LiCl was maintained at a 1 mL/min flow rate with an isocratic pump. A RID detector was set at 40 °C. The calibration with PSS ReadyCal-kit Poly(methyl methacrylate) and PSS WinGPC UniChrom^®^ Software 8.3SR2 was used for the M_w_ estimation.

In the presented research, an automatic sampler was used. Therefore, the data presented here is not directly comparable in terms of absolute values with the data previously published [16]. As such, the mock-ups kept in the freezer for seven months were considered as a reference for the moderately aged condition before storage (yellow bars in the figure in Section 3.2) because they presumably were the least influenced by degradation within that time and it was impossible to age new mock-ups artificially within the project. Instead, unaged mock-ups were remeasured with the newly installed autosampler (green bars in the figure in Section 3.2). The data intervals in the green and yellow bars should be considered as trends rather than the absolute values of M_w_ for the discussion and interpretation of data.

### 2.7. Gel Permeation Chromatography (GPC) for Determining the Camphor Content

An amount of 5 mg of the sample was dissolved in 1 mL of tetrahydrofuran (dried and fresh distilled from technical THF) and left to dissolve for 24 h in a shaker. Before injection, samples were filtered through a polytetrafluoroethylene filter (PTFE, pore size 1.0 μm, diameter 25 mm, VWR) to remove insoluble material and impurities. Then, 20 μL of sample solution was injected by an autosampler into the GPC system (SECcurity2, PSS, Mainz, Germany). This system was equipped with a set of PSS SDV columns (pre-column 3 μm 8 × 50 mm, 1 column 3 μm 10,000 Å 8 × 300 mm, and 2 columns 3 μm 1000 Å 8 × 300 mm) all maintained at 25 °C. The eluent THF was maintained at a 1 mL/min flow rate with an isocratic pump. A RID detector was set at 25 °C. The calibration with pure Camphor (Sigma-Aldrich) was done by a 5-point concentration calibration.

### 2.8. Sampling Strategy Bulk Analysis

Next, 3D-CN mock-ups were sampled using the same sampling strategy and position introduced in Elsässer et al. 2023 [16]. This bulk analysis considers the whole cross-section of a mock-up.

Four mock-ups of each geometry per storage condition were sampled. In order to cover a statistical set, at least two microsamples for each mock-up were considered. IC and GPC were performed within three months after storage.

### 2.9. Sampling Strategy for Depth Profile Analysis

**Figure 2:**
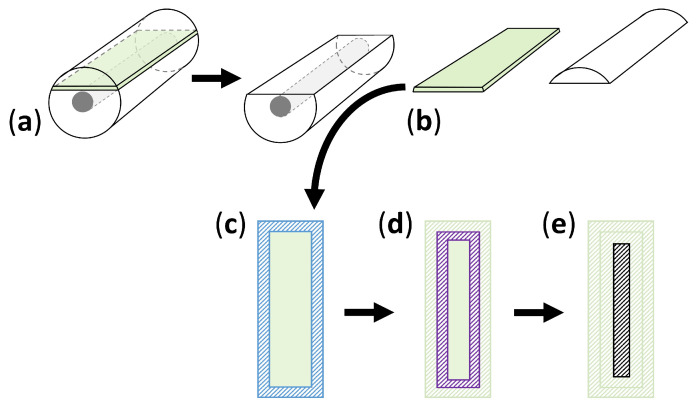
Sampling scheme for the profile analysis of the cylinders (dimensions: 30 mm (L); Ø = 10 mm). The greenish area indicates the position of the around 0.5 mm thick slice, which was used for the surface depths profile analysis (**a**). Therefore, the cylinder was sectioned breadthwise manually with a saw. The resulting slice (**b**) was then further segmented into the surface (**c**, blue hachure), 2 mm depths (**d**, purple hachure), and core (**e**, black hachure).

In order to chart the spatial distribution of the nitrogen content, camphor content, and M_w_ in the cylinders along their thickness, samples were collected at three different depths. A ca. 0.5 mm thick slice was taken just short above the metal core by sectioning the cylinder breadthwise and dividing it into three parts with a saw to guarantee reproducible sampling. An exemplary scheme of the cutting and area definition in depth is depicted in Figure 2. At least three microsamples of each depth were analyzed with IC and GPC.

### 2.10. Naturally Aged Objects for Investigation of the Depth Profile

**Figure 3:**
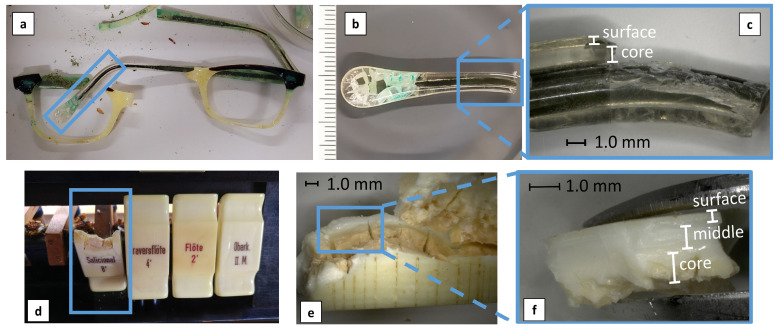
Original eyeglass temple before sampling (**a**); detail of this eyeglass temple displaying a heterogenous degradation, while the left ending is cracked, crazed, and greenish corrosion products of the metal core are visible, the right seems still intact and transparent (**b**); magnification of sampling points and depths of an intact area (**c**). The original position of the organ key (**d**); detail of the organ key showing a cracked and reddish discoloration in the core, while the surface seems still intact (**e**); magnification of the sampling positions (**f**).

Two historical artifacts were selected for the depth profile analyses. The first was a transparent eyeglass temple from the 1950s/1960s (Figure 3a–c), formerly belonging to the optical collection of the DM. However, those objects were removed from the inventory due to their advanced state of alteration. The second case study was a key former part of an organ situated in a Bavarian church (Figure 3d–f), and similar to the previous case, it was taken away due to its severe state of degradation.

Both objects show the characteristic heterogeneous nature of celluloid degradation (Figure 3), as described in the Introduction. The eyeglass’s visual appearance is non-uniform, particularly along the width, depicting areas visibly degraded and cracked while others seem still intact (Figure 3b). On the contrary, the organ key mainly depicts degradation gradients in depths, with a degraded and cracked core against an intact surface (Figure 3d,e).

The low material thickness around the metal core of the eyeglasses did not allow the separation of layers in regions with severe physical damage, such as cracks or crazes. Therefore, the samples for the depth profiling result from a spot that macroscopically appears intact (Figure 3c).

For sampling, a custom-made micro-tool was used to scrape off layers. While the eyeglass temple could be only sectioned into two layers (surface and core) (Figure 3b), the thickness of the organ key allowed three sampling depths (surface, middle, core) (Figure 3f), following its visual gradient of degradation.

## 3. Results and Discussion

### 3.1. Off-Gassing Monitoring with A-D Strips

**Figure 4:**
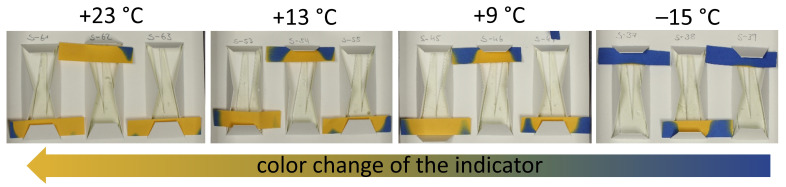
Detail of the mounted tines in archival boxes after seven months of storage. As a general tendency, the color change of the indicator paper was less at –15 °C than at the other storage temperatures.

Even though monitoring acid vapors through A-D strips is not a strictly quantitative method, the color change of the indicator papers after storage suggests that minor acid emissions occurred when stored at −15 °C rather than at +13 °C and +23 °C (Figure 4). Measurements with IC and GPC confirmed this tendency that the freezer condition preserved the mock-ups more effectively (Section 3.2 and Section 3.3). 

### 3.2. Bulk Analyses of Moderately Aged Sheets, Tines, and Cylinders

**Figure 5:**
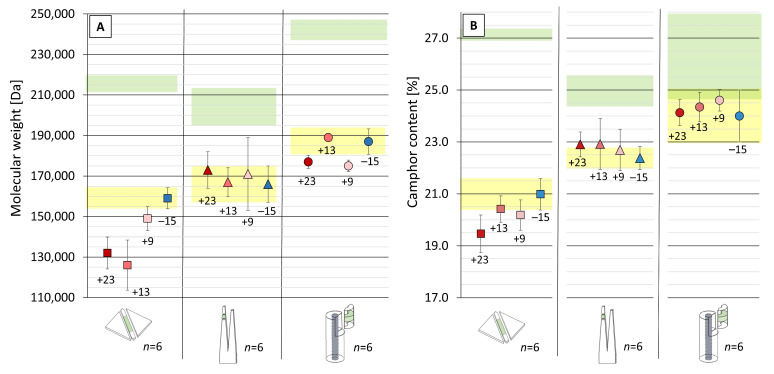
Bulk analyses of molecular weight (**A**) and camphor content (**B**) for the mock-ups (□ sheets, Δ tines, o cylinders) after seven months of storage at different temperatures. The green and yellow areas display the range of average values of M_w_ and camphor contents corresponding to unaged and aged mock-ups before storage, respectively. For more details, please consider Section 2.6.

After seven months, the M_w_ (Figure 5A) values and camphor contents (Figure 5B) of the sheets with 0.5 mm thickness at freezer temperature were the highest, reflecting the trend that the best preservation was obtained with the lowest temperature and a decrease of preservation happened with increasing temperatures. For the tines (depths: 2 mm) and cylinders (Ø: 10 mm), no trend could be recognized after the storage time when compared to the values after aging (yellow bar). The fact that only the sheets showed a clear trend can suggest that thicker mock-ups (tines and cylinders) might have become heterogeneous in profile. Under this assumption, bulk analyses would have averaged the material resulting from surface and core and flattened eventual differences in their chemical data and, therefore, the effect the tested storage temperatures might have had on their profile. In such a case, the thicker mock-ups would probably require longer exposure times (e.g., years instead of seven months of natural aging at room temperature) to detect the effect of storage temperatures with bulk sampling techniques. As written in the Introduction, degradation gradients can develop in the width and depth along the aging of 3D-CN [5,6,13]. Since this project had a concise time frame, the next part of this study involved profile analyses of the cylinders (the thickest produced mock-up, Ø: 10 mm) to check if degradation gradients occurred and try to assess the temperature’s effect on the cylinders’ preservation after seven months of storage.

### 3.3. Depth Profile of the Cylinders Moderately Aged

Since the sampling strategy adopted here considers the profile by collecting samples at different depths, the absolute data obtained in this section cannot be compared with published data by the authors, which used the bulk sampling technique [16]. 

**Figure 6:**
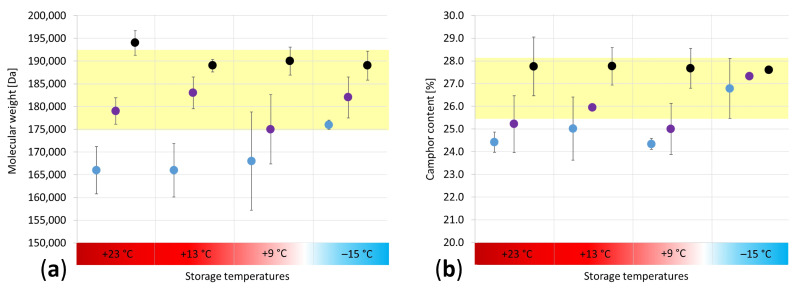
Depth profile analyses of the molecular weight distribution (**a**) of the camphor content (**b**) for the cylinders after seven months of storage at four different temperatures (blue = surface, violet = 2 mm depths, black = core). The yellow area marks the values achieved at −15 °C between the surface and core.

**Figure 7:**
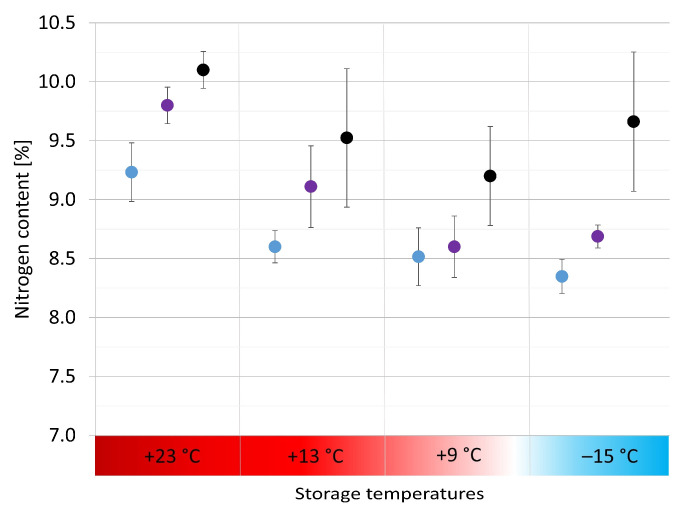
Calculated nitrogen content in depth profile (blue = surface, violet = 2 mm depths, black = core) after seven months of storage at four different temperatures.

For the M_w_ distributions, a trend is evident (Figure 6a), highlighting a better-preserved core than the surface at all storage temperatures. In addition, the results denote a more significant difference in the degradation condition along the depth at RT (+23 °C), +13 °C and +9 °C than at −15 °C, being characterized by a higher dispersion of the average values (beyond the yellow bar). The camphor content (Figure 6b) follows the same tendency as the M_w_, thus proving that the freezer storage is the most suitable. It can be deduced that the camphor sublimation was likely hindered at −15 °C, promoting, in turn, the physical preservation of the mock-ups. The positive effects of −15 °C on preserving the M_w_ and camphor content after seven months of storage were recognized by bulk measurements of the sheets (see Section 3.2) and depth profile analyses of the cylinders. However, this effect could not be detected for the nitrogen content of the cylinders in the depth profile (Figure 7). Only a gradient at RT with a better-preserved core than the surface was perceivable.

The lack of a trend in the nitrogen content is complex to interpret, as denitration comprises celluloid’s first and second degradation pathways [6]. Even though IC is undoubtedly an exact method enabling quantifying NO_2_^−^ and NO_3_^−^ in the range of a few ppm, it can be considered the least selective analytical technique applied in this study to follow celluloid’s deterioration through denitration because the distinction between anions molecularly bonded to the main polymer chain and those from the acids (decay products) is not possible. This circumstance might make tracing tiny differences in the nitrogen content challenging after seven months of storage. In addition, due to organization issues, IC analyses were the last to be conducted, which means that the mock-ups were kept at RT in the lab for three months after the end of storage, which might have led to material changes that could have influenced the results. Further studies with longer exposure times and shorter time intervals after concluding the storage and starting a measurement campaign are recommended in the future to avoid interferences with the analytical results.

### 3.4. Depth Profile of Severely Aged Historical Artifacts

**Figure 8:**
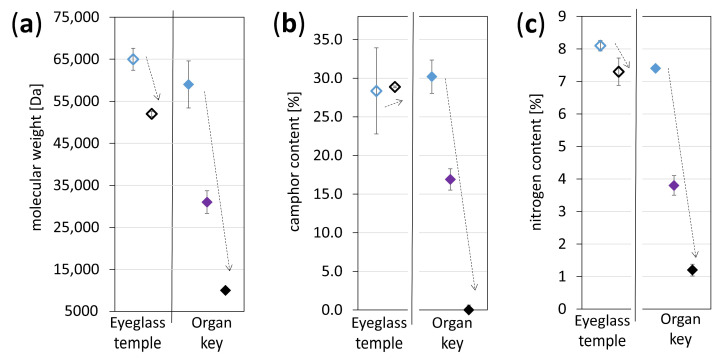
Molecular weight (**a**), camphor (**b**), and nitrogen (**c**) contents of the naturally aged objects sampled in depth profile (black = core, violet = middle, blue = surface). Dashed arrows indicate trends.

Figure 8 presents the degradation gradients developed in severely naturally aged artifacts in profile. Both objects present a similar trend with a better-preserved surface than the core. The camphor content of the eyeglass temple is characterized by a relatively high SD, making its interpretation not straightforward, while the organ key shows a clear drop in the camphor content towards the core (Figure 8b). The differences between the surface and the core are more substantial in the organ key, which matches its visual appearance, transiting from an intact surface to a cracked and discolored core. The typical reddish-brown color of the core indicates the local formation of NO_x_ species, pointing towards denitration (Figure 3d–f).

**Figure 9:**
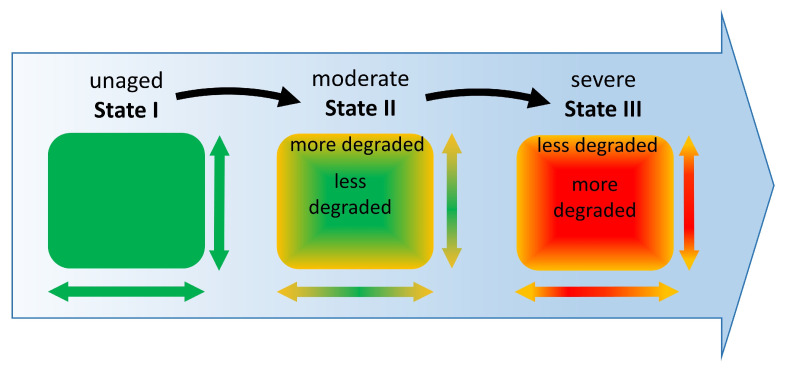
Possible schematic evolution of 3D-CN cross-sections with increasing aging, showing degradation gradients along the depth and width. In naturally aged objects several regions may have different conditions and gradients simultaneously.

Chavez Lozano et al. [6] investigated the same organ key and similar eyeglass temples following their molecular changes in profile with Fourier transform infrared spectroscopy (FTIR). The differences in the absorbance intensities for nitro (NO and NO_2_) and carbonyl (C=O) groups were followed along the depths. The IR absorbance intensities of nitro groups diminished from surface to core [6], matching the IC nitrogen content decrease in this study (Figure 8c). Chavez Lozano et al. [6] attribute the observed rise in the intensity of carbonyl groups towards the core to the formation of carbonyl-containing degradation products [18,50,51] rather than an increase in camphor content. The present study confirms this hypothesis for the severely degraded organ key (Figure 9) as the core’s camphor content decreases to zero, proving that the detected carbonyl groups result from degradation products. In addition, carbonyl formation detected by [6] indicates the creation of gluconolactone as the primary degradation product [51], formed through main chain scission through cleavage of the β-glycosidic bond. Figure 8a corroborates high losses in the M_w_ due to main chain scissions.

### 3.5. Evolution of 3D-CN Degradation Gradients in Depth Profile

As a general assumption, extrinsic factors (e.g., light, moisture, and heat) induce aging on plastics by interacting first with their surface (during processing and service), leading to degradation gradients characterized by more damaged outer than inner layers. The naked eye might not perceive the decay at the surface of plastic artifacts in moderate conditions, but in severe conditions, the plastic’s surface will appear clearly more damaged than the core. In contrast to all other plastics, 3D-CN objects can degrade with another path. After a more degraded surface than the core, as for all plastics in moderate conditions (state II), severely degraded 3D-CN objects are visibly identifiable by inner cracking and discoloration in the core with a much better-preserved surface. This depth profile is extremely peculiar to 3D-CN in state III [3,6,10,16,17,19] and unusual for other severely aged plastics. Such evolution of 3D-CN degradation gradients in a depth profile has not been exhaustively described yet, even though it has been extensively observed [3,6,10,16,17,19].

In two papers by the authors [6,16] 3D-CN mock-ups were processed (state I) and artificially aged until moderate and severe conditions. The mock-ups in states II and III looked similar to naturally aged 3D-CN objects [3,6,10,16,17,19], in their surface and profile, except for the occurrence of bubbles in the core (state III), which were never observed in real artifacts and for that reason the mock-ups in state III were excluded from this study. Analytical investigations by FTIR [6] could verify an incipient degradation but no gradient in moderate conditions, whereas a higher denitration in the core than at the surface was detected for state III. The later degradation gradient was also found in historical objects (state III) [6]. The present study improves the previous research [6] by investigating the depth profile of mock-ups in state II by liquid chromatographic methods. These techniques allowed for the first time to detect a gradient in a depth profile within the moderate condition (more degraded surface than core, Figure 6 and Figure 7). This gradient differs from the one analyzed in severe conditions [6], which at the core is characterized by more denitration, polymer chain scission, and reduction of camphor (Figure 8). The analyzed depth profiles of mock-ups in states II (data from this paper) and III (data from [6]) lead to the hypothesis that a transition in the orientation of the depth profile gradients must have occurred when evolving from the moderate to the severe condition. Assuming that state I is homogenous after production, the following scheme is suggested for representing the general evolution of degradation in the depth profile of 3D-CN objects (Figure 9).

Further studies are necessary to verify such transition along the degradation evolution and its causes, which should consider several decay mechanisms involving the plasticizers and the polymer itself. Since camphor sublimes already at RT [3,52], it likely leads to a concentration gradient throughout the material cross-section, which can influence the mechanical properties in profile and the respective material’s local response to environmental factors [53]. King et al. [53] state that two mass transfer steps of plasticizers occur: an internal diffusion from the bulk to the surface and an external mass transfer from the surface to the surroundings. In some cases, during aging, a change in the dominant step (“rate-limiting step”) can occur [53]. Both transport steps and eventual changes in their dominance might play a role in the transition of the depth profile gradients from state II to state III (Figure 9). Considering the degradation of the polymer, water molecules can penetrate the bulk more easily after camphor sublimation [10] and denitration [18]. Water molecules react with NO_x_ from the bulk, producing nitric acid there [3,10], giving a higher polar character to the matrix [18]. The entrapped acid may react with the polymer chains [2,18,19], enhancing further scission and cracks in the bulk [3], leading to a more degraded core, which is typical of 3D-CN in state III (Figure 9).

## 4. Conclusions

This study successfully investigated the effectiveness of four different storage temperatures (+23 °C, +13 °C, +9 °C, −15 °C) on the preservation of 3D-CN mock-ups in moderate conditions. The depth profile analyses, based on a tailored sampling strategy developed by the authors, were essential to characterize the extent of degradation gradients in the thick cylindric mock-ups. As a result, the effects of different storage temperatures could be assessed already after seven months. The storage temperature at −15 °C was the best preservation solution, keeping the M_w_, camphor, and nitrogen contents stable based on the chemical assessment. These results should be validated after a longer exposure time for all geometries. Such future studies should use the strategy developed in this paper and include not only physicomechanical testing to understand the celluloid behavior induced by freeze temperatures more comprehensively but also calculate the carbon footprint and energy consumption of the tested storage temperatures. 

The results strengthened the theory that a change in the spatial orientation of the degradation gradients occurs between moderate, i.e., more degraded outside than inside (outside-in), and severe, i.e., higher degradation extent inside than outside (inside-out), conditions for 3D-CN objects. This study highlights that the occurrence of degradation gradients better describes the complex alteration of 3D-CN, which was observed in severely naturally aged historical objects (state III) and thick-walled mock-ups in moderate conditions (state II), even though this latter showed no physical damage by the naked eye.

The heterogeneous nature of celluloids’ degradation represents a challenge for conservators because not one condition is present but rather a range of condition states, which are not easy to summarize in one category. In addition, conservation scientists should pay attention to the fact that chemical and/or mechanical information obtained from a measured location or sample is not representative of the whole object but specific to the analyzed region and, depending on the condition state, likely a degradation gradient with a specific spatial orientation is present.

The chemical assessment of this study implemented a multi-analytical approach for monitoring the three main decay processes of celluloid, which may occur parallel. Among the decay process investigated, the M_w_ was the most sensitive parameter to follow changes after artificial aging [16] and seven months of storage. As such, GPC can be in general considered particularly adequate to follow the degradation of 3D-CN objects. Future research should consider the depth profiling strategy to study celluloid degradation to assess its heterogeneous nature and the effectiveness of preservation solutions.

## Figures and Tables

**Table 1:** Materials used in the enclosure of mock-ups.

Materials	Functions	Details
acid-free archival boxes (Stülpschachtel “Loreley”—DIN A6 Premium, Hans Schröder GmbH, Karlsdorf-Neuthard, Germany)	stability defined volume humidity buffer acidic vapors absorber	PAT tested acid free calcium carbonate buffered blue-grey 1.00 mm solid board
archival mat board (CHRONOS Fotoarchivkarton—DIN A6—300 g/m^2^, Hans Schröder GmbH, Karlsdorf-Neuthard, Germany)	organization of mock-ups in the boxes padding material humidity buffer acidic vapors absorber	PAT tested acid free unbuffered natural white 300 g/m^2^
A-D strips (Danchek acid detection test strips, Long Life for Art, Eichstetten, Germany)	acidic vapors monitoring (knowing that their response will be reduced below room temperature)	paper strips dye-coated with Bromocresol green indicator
data-logger (testo 174 H—temperature and humidity mini data-logger, Testo SE & Co. KGaA, Lenzkirch. Germany)	T and RH monitoring	range (T): −20 to +70 °C range (RH): 0 to 100% RH
sealable barrier film (ESCAL NEO Folie, Long Life for Art, Eichstetten, Germany)	hinders the exchange of oxygen and water vapor creates a uniform micro-environment at the beginning of the experiment prevents water condensations on the mock-ups T and humidity buffer	ceramic-coated PET/PE film film thickness: 115 μm oxygen permeation: <0.1 cm^3^/m^2^/d/atm (25 °C, 60% RH) vapor permeation: 0.08 g/m^2^/d (40 °C, 90% RH)

T—temperature; RH—relative humidity; PAT—Photographic Activity Test; PET—polyethylene terephthalate; PE—polyethylene.

## Data Availability

Data available on request.

## Supplementary Materials

The following supporting information can be downloaded at: https://www.mdpi.com/article/10.3390/polym15204056/s1.
