# Supplementary material for: Frozen, Cold, or Cool? Chemical Assessment of the Effectiveness of Storage Conditions for Celluloid 3D Objects
Source: Polymers (Basel). 2023 Oct 11;15(20):4056. doi: 10.3390/polym15204056 (PMC10609767; doi:10.3390/polym15204056)
Supplement: Supplementary file 1 [file polymers-15-04056-s001.zip › polymers-2568082-supplementary.pdf]

polymers

Supplementary information

Frozen, Cold, or Cool? Chemical Assessment of the Effectiveness of Storage Conditions for Celluloid 3D Objects

Christina Elsässer [1,2], Eva Mariasole Angelin [1], Peter Montag [3], Harald Hilbig [4], Christian U. Grosse [2] and Marisa Pamplona [1,*]

1 Conservation Science Department, Deutsches Museum, Museumsinsel 1, 80538 Munich, Germany; c.elsaesser@deutsches-museum.de (C.E.); e.angelin@deutsches-museum.de (E.M.A.)
2 Chair of Non-Destructive Testing, Technical University of Munich, Franz-Langinger-Straße 10, 81245 Munich, Germany; grosse@tum.de
3 PSS a Part of Agilent, Polymer Standards Service GmbH, In der Dalheimer Wiese 5, 55120 Mainz, Germany; pmddf@web.de
4 Professorship of Mineral Construction Materials, Technical University of Munich, Franz-Langinger-Straße 10, 81245 Munich, Germany; harald.hilbig@tum.de
* Correspondence: m.pamplona@deutsches-museum.de

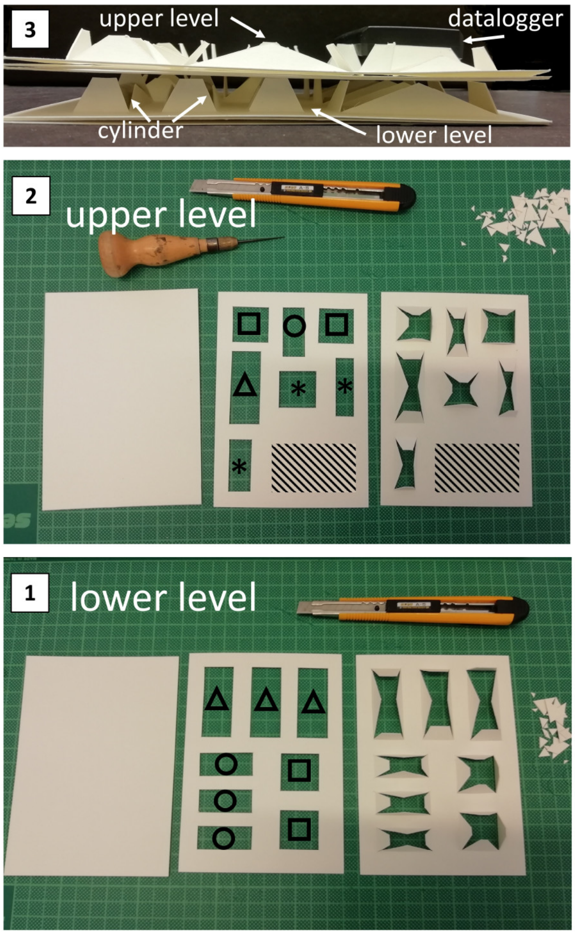

Figure S1. Preparation of the mat board structure for the storage boxes. Each storage box hosted four mock-ups per geometry distributed on a lower level (1) and an upper level (2). Both levels were mounted on top of each other (3) and successively placed in the storage boxes. Symbols indicate a place for: □ sheet, Δ tine, o cylinder, * fragment of an object (not investigated in this study). The hatchures point out the place for a datalogger.

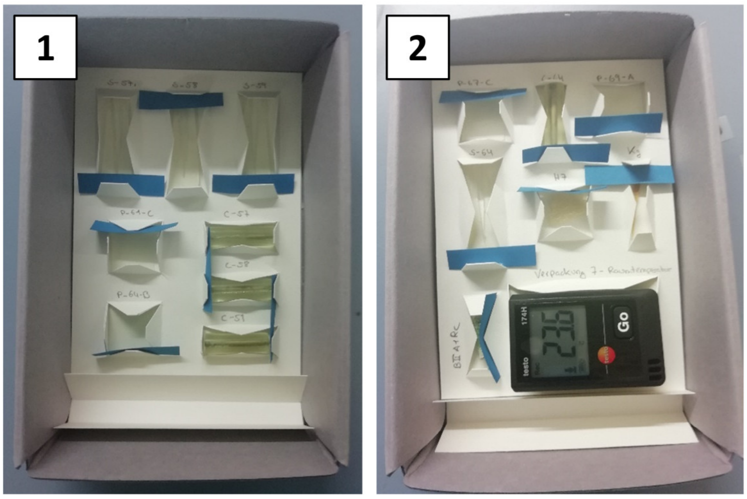

Figure S2. Lower level of the mat board structure mounted in the storage box and with mock-ups and indicator papers (1). Storage box after mounted the upper level and equipping it with mock-ups, indicator papers and a datalogger for temperature and relative humidity (2).

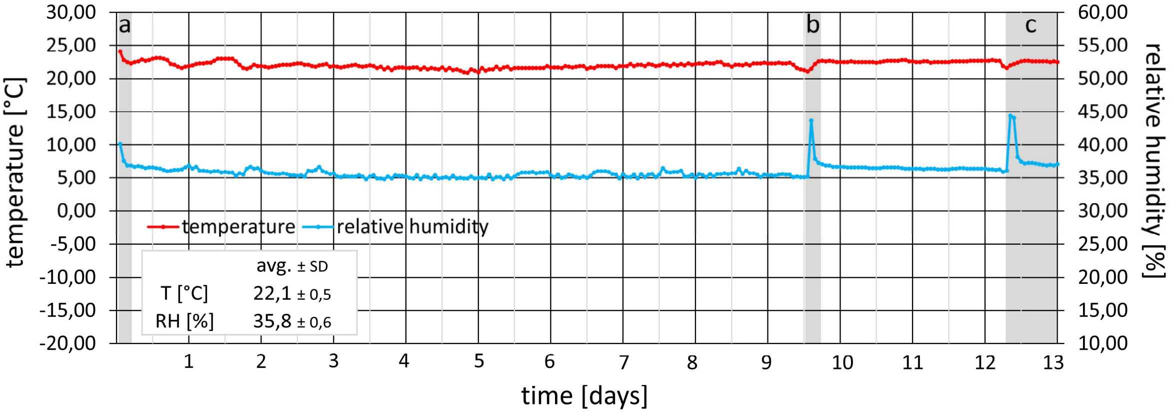

Figure S3. Temperature and relative humidity in the desiccator while preconditioning the mock-ups and packaging materials. After 12 days of exposure, the mock-ups were packed. The gray areas in the graph highlight the acclimatization phase at the beginning (a), an opening of the exsiccator for maintenance purposes (b), and the opening of the exsiccator at the end of the preconditioning (c). The grey areas were not considered in the calculation of the temperature and relative humidity averages.

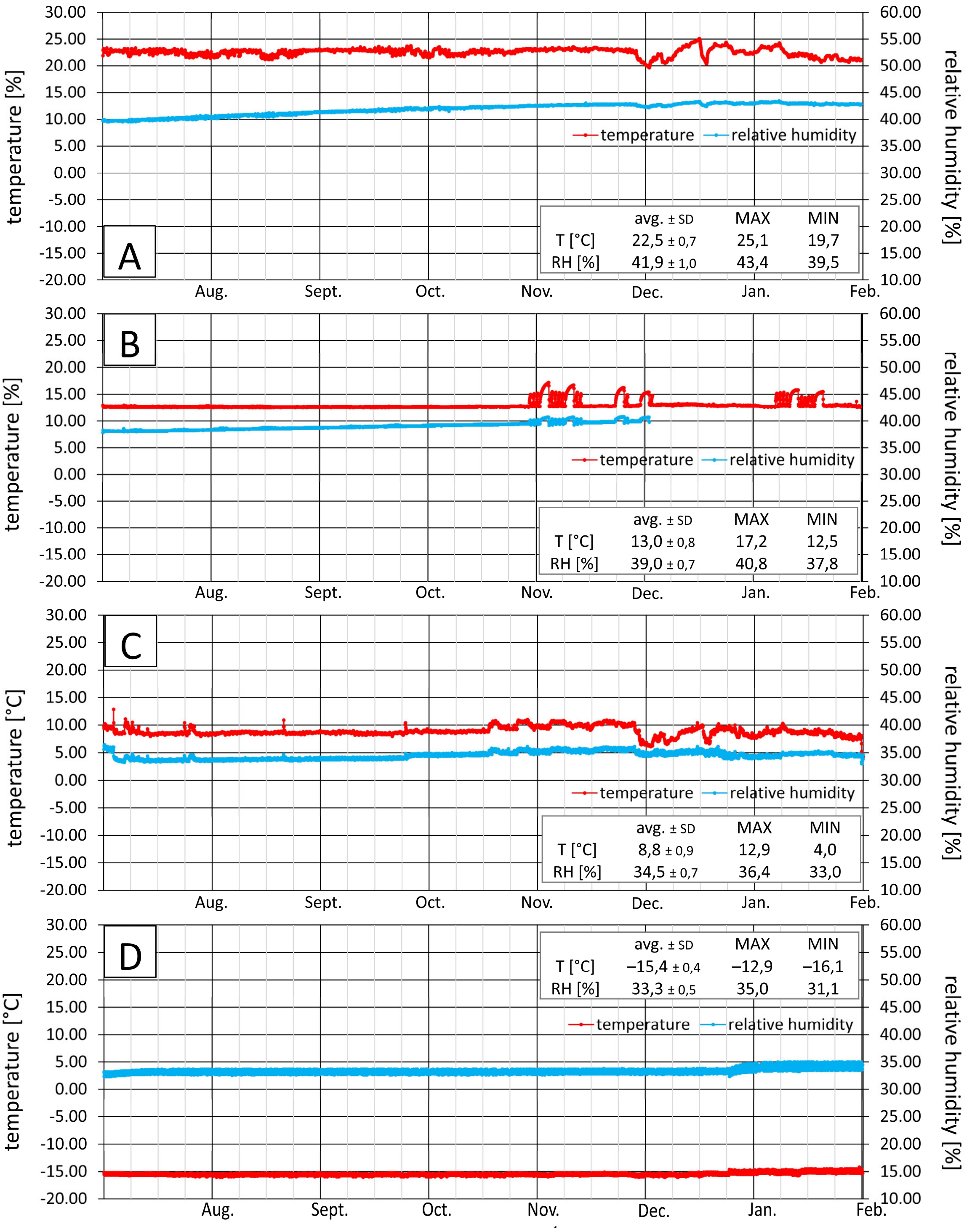

Figure 4. recorded climate data of the data logger enclosed in the packaging system during the seven months of exposure in 2021/2022: A, ambient temperature (reference); B, cool temperature (controlled by air-conditioning system); C, cold temperature (controlled by a fridge); D, frozen temperature (controlled by a freezer). For each scenario the average (avg.), the maximum (MAX) and minimum values (MIN) for temperature (T) and relative humidity (RH) are reported.

Note: For B: The recording in the enclosure accidentally ended short after five months of exposure. Due to this, the recorded temperature data of another datalogger, which was placed inside the thermos box together with the packed samples, was used, but no data related to the relative humidity enclosed in the packaging system were available.
